# Psychological Correlates of Self-Harm Ideation Among Depressive Outpatients: The Roles of Rumination and Depressive Symptom Severity

**DOI:** 10.3390/bs16101943

**Published:** 2026-10-19

**Authors:** Barbara Chuen Yee Lo, Linshan Du, Lucy B. G. Tan

**Affiliations:** 1 School of Educational Psychology and Inclusion, Faculty of Education, Monash University, Clayton, VIC 3800, Australia; linshan.du@monash.edu; 2 Clinical Psyhology, School of Social and Health Sciences, James Cook University, Singapore Campus, 149 Sims Drive, Singapore 387380, Singapore; lucy.tan@jcu.edu.au; 3 School of Psychology, University of Aberdeen, King’s College, Aberdeen AB24 3FX, Scotland, UK

**Keywords:** depression, rumination, self-harm ideation

## Abstract

Self-harm is a prevalent and clinically significant concern among individuals experiencing depressive symptoms. However, differences in psychological profiles between depressive outpatients with and without self-harm ideation remain poorly understood. To address this gap, the present study examined emotional and cognitive factors associated with self-harm ideation among depressive outpatients. A total of 164 depressive outpatients with a current or past major depressive episode were recruited from public hospitals in Hong Kong. Participants completed standardized self-report measures assessing self-harm ideation, depressive and anxiety symptoms, and rumination. Compared with those without self-harm ideation, individuals reporting self-harm ideation exhibited significantly greater symptom severity, higher rumination, and more pronounced negative affect. Rumination was positively associated with self-harm ideation, whereas the association with reflective thinking was not statistically significant. Depressive symptom severity moderated the relationship between rumination and self-harm ideation, such that the association was stronger at lower levels of depressive symptoms. These findings suggest that distinct emotional and cognitive profiles characterize depressive outpatients who report current self-harm ideation and that the association between rumination and self-harm ideation may vary according to depressive symptom severity.

## 1. Introduction

### 1.1. Depression and Self-Harm Ideation

Self-harm refers to any non-fatal, intentional act of self-injury, irrespective of method or suicidal intent (Geulayov et al., 2022; Hawton et al., 2003). It represents a major public health concern and is strongly associated with suicide risk. Globally, self-harm accounts for an estimated 14 million episodes annually and is a robust predictor of both subsequent suicide and repeated self-harm (Chan et al., 2016; Larkin et al., 2014; Moran et al., 2024). Distinct from enacted self-harm, self-harm ideation refers to thoughts about harming oneself, with or without suicidal intent, and is distinct from the enactment of self-harm behavior (Giallo et al., 2018; Nielsen et al., 2017). This distinction is important because thoughts of self-harm do not necessarily lead to self-harm behavior, and the factors associated with ideation may differ from those associated with enactment (Etherson et al., 2025; Nielsen et al., 2017). Depressive disorders are among the psychiatric conditions most strongly associated with self-harm and suicidality, including suicidal ideation and attempts (Cai et al., 2021; Dong et al., 2019; Hawton et al., 2013). More specifically, depressive symptoms and psychological distress have been associated with self-harm ideation across clinical and community populations (Giallo et al., 2018; Humber et al., 2013; Hovaldt et al., 2022).

Although depressive symptoms have been associated with self-harm ideation, such thoughts are not reported by all individuals with MDD (Zhang et al., 2026). The variability suggests that depressive symptoms alone are insufficient to explain self-harm ideation. Contemporary models of self-harm emphasize the importance of identifying psychological processes that may explain why some individuals respond to distress with maladaptive behaviors whereas others do not (Chapman et al., 2006; Selby et al., 2008). In particular, cognitive processes involved in responses to negative emotional experiences may help explain why some individuals report self-harm ideation whereas others do not.

### 1.2. Emotion Regulation Models of Self-Harm

In the literature, a growing body of theoretical and empirical work conceptualizes self-harm as a maladaptive strategy for regulating intense emotional states (Chapman et al., 2006; Klonsky, 2007). Within this perspective, self-harm is understood not merely as an impulsive act but as a behavior that temporarily alleviates overwhelming psychological distress (Nock & Prinstein, 2004). The Experiential Avoidance Model (EAM) for example proposes that individuals may engage in self-harm to escape or reduce aversive emotional experiences (Angelakis & Gooding, 2021; Chapman et al., 2006). In accordance with this model, when distress becomes intolerable and individuals perceive limited alternatives for coping, self-harm may function as a negatively reinforced behavior that provides short-term emotional relief, thereby increasing the likelihood of repeated engagement (Gratz et al., 2018; Rodham et al., 2004).

An extension of this perspective is offered by the Emotional Cascade Model (ECM), which postulates that self-harm may arise from escalating cycles of emotional and cognitive processes (Selby et al., 2008). Within the framework of this model, stressful experiences initially trigger negative emotional states, which may then be intensified and prolonged through repetitive negative thinking, particularly rumination (Selby et al., 2009; Selby et al., 2013). As individuals repeatedly focus on distressing thoughts and feelings, emotional arousal becomes increasingly amplified, creating what has been described as an “emotional cascade” (Selby et al., 2008). During such cascades, distress may reach a level that becomes extremely difficult to tolerate. Self-harm may then emerge as a behavioral strategy to interrupt or distract from the escalating cycle of rumination and emotional distress (Selby et al., 2008; Klonsky, 2007). In this way, cognitive processes that maintain negative affect are central mechanisms through which emotional distress may translate into self-harm. Although the EAM and ECM primarily explain the enactment of self-harm, they provide a conceptual basis for examining cognitive processes associated with self-harm ideation.

### 1.3. Rumination and Self-Harm

One cognitive process that has received extensive attention in depression research is rumination. The well-known Response Styles Theory defines rumination as a repetitive and passive focus on the causes and consequences of one’s negative emotional state (Nolen-Hoeksema, 1991). Rather than facilitating adaptive coping or problem solving, ruminative thinking tends to maintain negative mood, prolong emotional distress, and interfere with effective emotion regulation (Nolen-Hoeksema et al., 2008). Individuals who engage in high levels of rumination often become trapped in cycles of repetitive negative thinking that intensify feelings of sadness, hopelessness, and self-criticism (Treynor et al., 2003).

Rumination is therefore particularly relevant within the framework of the ECM. Repetitive negative thinking may amplify emotional reactions to stressors, creating feedback loops in which rumination intensifies distress, and distress further triggers additional rumination (Selby et al., 2008; Selby et al., 2013). Previous research has linked higher levels of rumination to greater depressive symptom severity, suicidal ideation, and self-harming behaviors (Borrill et al., 2009; Marroquín et al., 2010). Individuals who engage in self-harm frequently report elevated levels of repetitive negative thinking and maladaptive coping strategies, suggesting that rumination may contribute to the escalation of emotional distress that precedes self-harm (Nicolai et al., 2016). However, self-focused attention is not a unitary construct, and a distinction has been drawn between two conceptually distinct forms of self-focus: rumination and reflection. Trapnell and Campbell (1999) for example defined rumination as a recurrent focus on distress, perceived threats, losses, and injustices to the self, whereas reflection was conceptualized as a more purposeful and curiosity-driven form of self-attention. These two forms of self-focus appear to have different psychological correlates. Rumination has been more closely linked to maladaptive emotional functioning, whereas reflection has shown less consistent associations with maladaptive emotional outcomes (Joireman et al., 2002). In a high-risk clinical sample, reflection was not significantly associated with concurrent suicidal ideation or a lifetime history of suicide attempts, nor did it show significant prospective associations with either outcome (Horwitz et al., 2019). In the present study, these two forms of self-focus were examined using the Rumination–Reflection Questionnaire (Trapnell & Campbell, 1999), which was specifically developed to distinguish rumination from reflection. Examining these processes separately may therefore help clarify whether rumination and reflection are differentially associated with self-harm ideation.

### 1.4. The Role of Emotional Context in Rumination and Self-Harm Ideation

Although rumination is widely considered maladaptive, its association with self-harm ideation may vary depending on the broader emotional context. Rumination can amplify and prolong distress by sustaining attention to negative thoughts and emotions (Nolen-Hoeksema et al., 2008). According to the ECM, rumination and negative affect may become mutually reinforcing, contributing to escalating emotional distress and dysregulated behaviors (Selby et al., 2008; Selby et al., 2013). Prospective evidence has also indicated that rumination and negative affect interact in relation to subsequent engagement in non-suicidal self-injury and its frequency (Nicolai et al., 2016). Although this evidence primarily concerns self-injury behaviors rather than self-harm ideation, it provides a rationale for considering whether the association between rumination and self-harm-related outcomes varies according to emotional context.

Given the close relationship between depressive symptoms and negative affect, depressive symptom severity may represent a clinically relevant contextual factor in the association between rumination and self-harm ideation. However, existing evidence does not establish whether depressive symptom severity strengthens or weakens this association, and the ECM does not specifically predict the direction of this moderating relationship. Examining depressive symptom severity as a potential moderator may therefore help clarify whether the concurrent association between rumination and self-harm ideation differs across levels of depressive symptoms.

### 1.5. The Present Study

Despite growing evidence linking rumination to self-harm-related outcomes, relatively few studies have examined the distinct associations of rumination and reflection with self-harm ideation in clinical populations. Furthermore, the extent to which depressive symptom severity moderates the association between rumination and self-harm ideation remains unclear. To address these gaps, the present study examined the emotional and cognitive correlates of self-harm ideation among outpatients with a current or past major depressive episode. Drawing on Response Styles Theory and the ECM, the study focused on the distinct roles of rumination and reflection in relation to self-harm ideation, as well as the potential moderating role of depressive symptom severity.

The study had two primary aims. First, it sought to compare individuals with and without self-harm ideation in terms of depressive symptoms, psychological distress, rumination, and reflection. Second, it examined the associations of rumination and reflection with self-harm ideation and whether depressive symptom severity moderated the association between rumination and self-harm ideation.

Based on the theoretical and empirical literature reviewed above, two hypotheses were proposed. First, individuals with self-harm ideation were expected to report higher levels of depressive symptoms, psychological distress, and rumination than those without self-harm ideation. Second, rumination was expected to be positively associated with self-harm ideation, whereas reflection was expected to show little or no association. In addition to these hypotheses, the study examined whether depressive symptom severity moderated the association between rumination and self-harm ideation. Given the limited theoretical and empirical evidence regarding the direction of this interaction, no specific directional prediction was proposed.

## 2. Materials and Methods

### 2.1. Participants

Participants were recruited from public hospitals in Hong Kong, and a total of 165 outpatients took part in the study. One participant was excluded due to missing information on self-harm ideation, resulting in a final sample of 164 participants. The sample comprised 136 females (82.9%) and 28 males (17.1%), indicating a marked gender imbalance. Participants were aged 19 to 65 years (*M* = 45.18, *SD* = 10.48). Eligibility was based on the presence of a current or past major depressive episode (MDE), assessed using the Structured Clinical Interview for DSM Disorders (SCID; First et al., 2016), rather than requiring a current diagnosis of major depressive disorder (MDD). Comorbid psychiatric conditions were permitted under the original eligibility criteria. Of the 164 participants included in the analysis, 122 had a recorded diagnosis of MDD. Participants were receiving outpatient psychiatric care at the time of recruitment. Information regarding antidepressant medication use was not available for the present analysis. The study protocol was reviewed and approved by the Joint Chinese University of Hong Kong–New Territories East Cluster Clinical Research Ethics Committee (Ref. No. CRE-2013.333), and the study was conducted in accordance with the Declaration of Helsinki and its subsequent amendments.

### 2.2. Measures

#### 2.2.1. Self-Harm

Self-harm ideation was assessed using Item 11 of the DSM-5 Self-Rated Level 1 Cross-Cutting Symptom Measure—Adult, which asks participants how much or how often they had been bothered by “thoughts of actually hurting yourself” during the past two weeks (American Psychiatric Association, 2013). Response options were rated on a 5-point scale ranging from 0 (none; not at all) to 4 (severe; nearly every day). For the primary analyses, responses to the five-category self-harm ideation item were dichotomized as absence (score = 0) or presence (score ≥ 1) of current self-harm ideation. This coding reflected the study’s primary focus on whether participants reported any current self-harm ideation, rather than differences across the original response categories. Because dichotomization discards information contained in the ordered responses, the original five-category outcome was also retained for a sensitivity analysis.

The broader DSM-5 Self-Rated Level 1 Cross-Cutting Symptom Measure—Adult has demonstrated acceptable psychometric properties in prior research, including evidence of internal consistency and construct validity (Bravo et al., 2018; Ma et al., 2021).

#### 2.2.2. Depression

Depressive levels were measured using the depression subscale of the Hospital Anxiety and Depression Scale (HADS-D) (Zigmond & Snaith, 1983). The HADS-D consists of seven items assessing depressive symptoms over the past week. Each item is rated on a 4-point scale from 0 to 3, yielding a total score ranging from 0 to 21. Scores were categorized as normal (0–7), borderline abnormal (8–10), or abnormal (11–21). The HADS-D was selected as the primary measure for classification because its cut-off score of 8 has been extensively validated as an indicator of elevated depressive symptoms, demonstrating an acceptable balance between sensitivity and specificity across clinical settings (Bjelland et al., 2002; Brennan et al., 2010). It also minimizes the assessment of somatic symptoms that may overlap with physical health conditions, making it suitable for hospital-based samples (Zigmond & Snaith, 1983). The HADS-D categories were used to describe the distribution of depressive symptom severity, whereas the continuous HADS-D score was used in the moderation analysis to preserve the full variability in depressive symptoms. The Chinese version of the HADS has demonstrated acceptable reliability and validity (Li et al., 2016). In the present study, the HADS-D showed good internal consistency (Cronbach’s α = 0.80).

#### 2.2.3. Depression, Anxiety and Stress

Broader emotional distress was assessed using the depression, stress and anxiety subscales of the 21-item Depression Anxiety Stress Scales (DASS-21) (Lovibond & Lovibond, 1995; Henry & Crawford, 2005). Each subscale contains 7 items. Participants rated the extent to which each statement applied to them over the past week. Each item was scored on a 4-point scale from 0 to 3. Subscale scores were calculated by summing item scores and multiplying by two, following the standard scoring procedure for the DASS-21 to align scores with the original 42-item version (Lovibond & Lovibond, 1995). Higher scores indicate greater levels of depression, stress or anxiety. Based on established cut-off scores, depression severity was classified as normal (0–9), mild (10–13), moderate (14–20), severe (21–27), or extremely severe (28 or above); anxiety severity was classified as normal (0–7), mild (8–9), moderate (10–14), severe (15–19), and extremely severe (20+)s; stress severity was classified as normal (0–14), mild (15–18), moderate (19–25), severe (26–33), and extremely severe (34+). The DASS-21 total and subscale scores were treated as continuous variables in the group-comparison analyses. The DASS-21 has demonstrated good psychometric properties, including satisfactory reliability, in prior research (Osman et al., 2012). In the present study, internal consistency was excellent for the DASS-21 total score (Cronbach’s α = 0.95) and the depression subscale (α = 0.90), and good for the anxiety (α = 0.85) and stress (α = 0.84) subscales.

#### 2.2.4. Worry

Trait worry was measured using the Penn State Worry Questionnaire (PSWQ) (Meyer et al., 1990). The PSWQ is a 16-item self-report scale designed to assess trait worry. Participants selected the option that best described how typical each statement was for them. Each item was rated on a 5-point Likert scale from 1 (not at all typical of me) to 5 (very typical of me). Total scores were calculated by summing all items, with scores ranging from 16 to 80. Higher scores indicate higher levels of trait worry. For descriptive purposes, PSWQ total scores were grouped into three levels: low worry (16–39), moderate worry (40–59), and high worry (60–80). The PSWQ has shown good psychometric properties in previous studies (Brown et al., 1992; Zhong et al., 2009). Internal consistency in the present study was high (Cronbach’s α = 0.89).

#### 2.2.5. Rumination and Reflection

The Chinese version of the Rumination–Reflection Questionnaire (RRQ), adapted from the original measure developed by Trapnell and Campbell (1999) and validated among Chinese university students by Yuan et al. (2010), was administered in the present study. The RRQ includes two subscales, rumination, and reflection, which assess two forms of self-focused thought. The rumination subscale consists of 12 items. Participants rated how well each statement described their typical thoughts on a 5-point Likert scale ranging from 1 (strongly disagree) to 5 (strongly agree). Rumination scores were calculated by summing all item responses, with total scores ranging from 12 to 60. Higher scores indicate higher levels of ruminative thinking. Negatively worded items in the rumination subscale (Items 5, 9, and 10) were reverse scored before analysis.

The reflection subscale also consists of 12 items. Participants rated how well each statement described their typical thoughts on the same 5-point Likert scale ranging from 1 (strongly disagree) to 5 (strongly agree). Reflection scores were calculated by summing all item responses, with total scores ranging from 12 to 60. Higher scores indicate greater reflective self-focus. Negatively worded items in the reflection subscale (Items 4, 7, 8, 9, and 10) were reverse scored before analysis.

In this study, rumination was treated as a continuous variable. Previous studies have provided evidence for the reliability and validity of the RRQ (Joireman et al., 2002), including its Chinese version (Yuan et al., 2010). In the present study, the RRQ rumination subscale demonstrated acceptable internal consistency (Cronbach’s α = 0.72), Boateng et al. (2018) noted that 0.70 is often regarded as an acceptable cutoff for reliability, although higher coefficients are preferred. The reflection subscale demonstrated good internal consistency (Cronbach’s α = 0.84).

### 2.3. Procedure

Before taking part in the study, all participants were informed of their rights, including the right to stop participating at any time without any negative consequences. Participation was voluntary, and this was clearly explained before data collection started. Participants were tested individually at a private meeting room in the hospital. Upon receiving informed consent, research assistant conducted the semi-structured interviews (i.e., SCID) and then asked participants to fill in a set of questionnaires. A clinical psychologist randomly observed the testing sessions to ensure procedural consistency. Participants were thanked and debriefed at the end of the session, which took approximately 2 h to complete.

## 3. Results

### 3.1. Differences in Demographic and Clinic Suggestal Characteristics by Current Self-Harm Ideation Status

The distribution of responses across the original five-category self-harm ideation item was as follows: 116 participants (70.7%) scored 0 (none), 19 (11.6%) scored 1 (slight), 16 (9.8%) scored 2 (mild), 7 (4.3%) scored 3 (moderate), and 6 (3.7%) scored 4 (severe). Percentages were calculated using the 164 participants with valid responses; one additional participant had a missing response and was excluded from the analytic sample. A total of 48 participants (29.3%) were classified as reporting current self-harm ideation, defined as a self-harm ideation score of 1 or higher, whereas 116 participants (70.7%) were classified as having no self-harm ideation, defined as a self-harm ideation score of 0. No significant group differences were found for gender, χ^2^(1) = 0.14, *p* = 0.714, or age, *t*(162) = 1.09, *p* = 0.280.

Consistent with the hypothesis, Table 1 shows that participants with current self-harm ideation reported higher levels of depressive symptoms than those without self-harm. HADS-D severity categories differed significantly between the two groups, χ^2^(2) = 26.33, *p* < 0.001, with a substantially greater proportion of participants in the self-harm group falling within the clinical range. Similarly, the self-harm group reported significantly higher DASS Depression scores, *t*(160) = 8.35, *p* < 0.001.

The current self-harm ideation group also reported significantly greater emotional distress. Compared with participants without current self-harm ideation, those with current self-harm ideation had higher scores on DASS Total, *t*(160) = 7.49, *p* < 0.001, DASS Anxiety, *t* (160) = 5.46, *p* < 0.001, and DASS Stress, *t*(160) = 6.39, *p* < 0.001. In addition, they reported higher levels of worry as measured by the PSWQ, *t*(119.79) = 3.40, *p* < 0.001.

Participants with current self-harm ideation also reported significantly higher RRQ Rumination scores than those without current self-harm ideation, *t*(132.89) = 4.07, *p* < 0.001. However, no significant group difference was found for RRQ Reflection, *t*(162) = 0.33, *p* = 0.744.

### 3.2. Associations of Rumination and Reflective Thinking with Current Self-Harm Ideation

To examine the second hypothesis, Spearman’s rho correlation analyses were conducted to assess the associations of rumination and reflective thinking with current self-harm ideation. As expected, Table 2 shows that self-harm ideation was significantly and positively associated with rumination, *r*_s_ = 0.268, *p* < 0.001, indicating that higher levels of rumination were related to greater self-harm ideation. In contrast, reflective thinking was not significantly associated with self-harm ideation, *r*_s_ = −0.041, *p* = 0.600. These findings supported the hypothesis that rumination, but not reflective thinking, was related to current self-harm ideation.

### 3.3. Moderating Effect of Depressive Symptoms on the Association Between Rumination and Self-Harm

**Figure 1:**
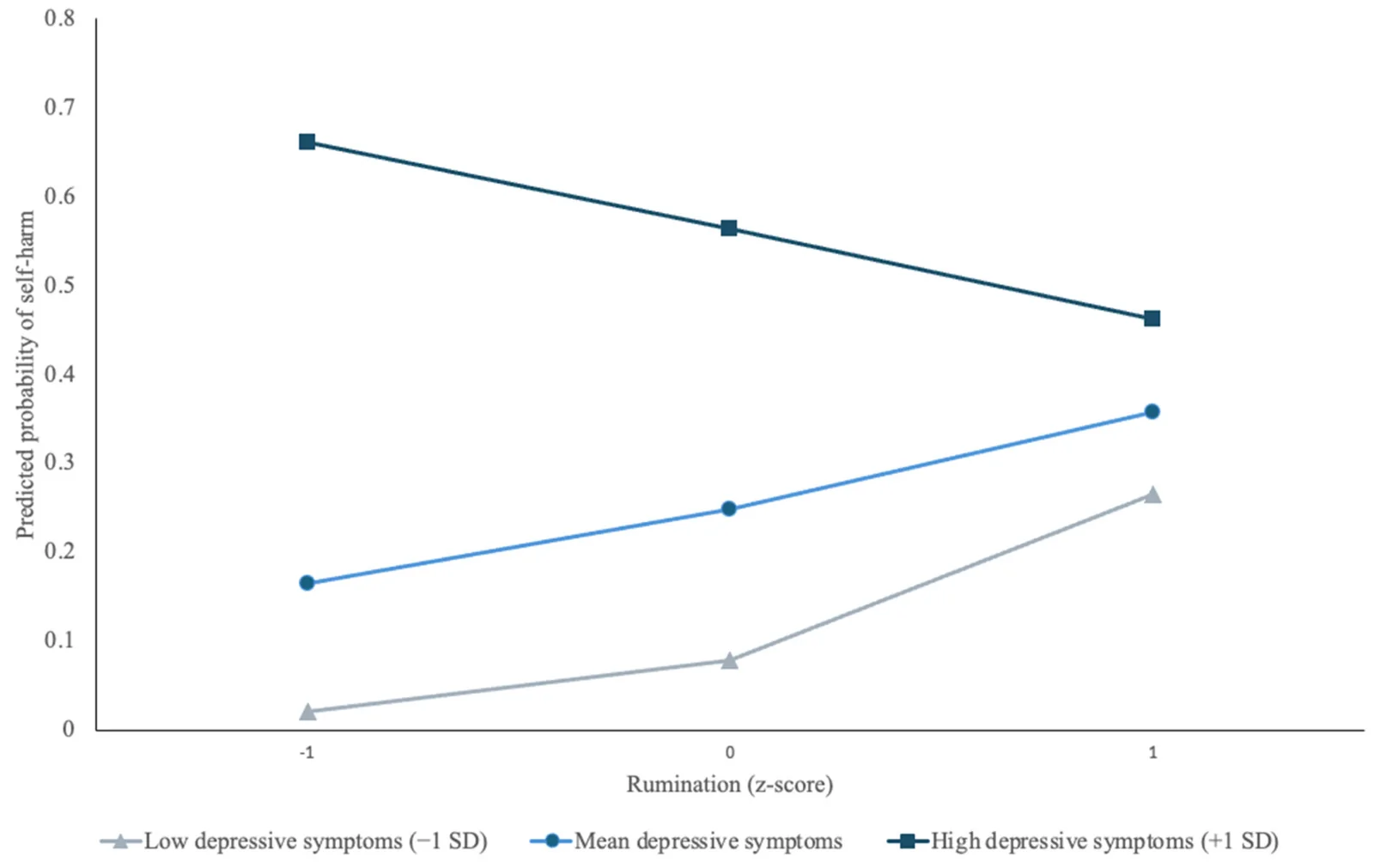
Predicted Probability of Self-harm Ideation as a Function of Rumination at Low, Mean, and High Levels of Depressive Symptoms. Note. Points represent predicted values of current self-harm ideation at low (−1 *SD*), mean, and high (+1 *SD*) levels of depressive symptoms across levels of rumination.

To test the null hypothesis, a binary logistic regression analysis was conducted. Prior to analysis, rumination scores from the Rumination–Reflection Questionnaire (RRQ) and depressive symptom scores from the Hospital Anxiety and Depression Scale–Depression subscale (HADS-D) were standardized to z scores. An interaction term was then calculated as the product of these two standardized scores. Current self-harm ideation was entered as the binary dependent variable, with standardized rumination as the focal predictor and standardized depressive symptoms as the moderator. Both standardized predictors and their interaction term were entered simultaneously into the model. The regression coefficients are presented in Table 3. The overall model was statistically significant, χ^2^(3) = 40.36, *p* < 0.001, accounting for 21.8% of the variance in self-harm based on Cox and Snell R^2^ and 31.1% based on Nagelkerke *R*^2^. The Hosmer–Lemeshow goodness-of-fit test was non-significant, χ^2^(8) = 4.00, *p* = 0.857, indicating that the model demonstrated an adequate fit to the data. Depressive symptoms significantly predicted higher self-harm ideation risk, *B* = 1.36, *SE* = 0.32, Wald = 18.51, *p* < 0.001, OR = 3.90, 95% CI [2.10, 7.24]. The main effect of rumination was marginal, *B* = 0.52, *SE* = 0.28, Wald = 3.40, *p* = 0.065, OR = 1.68, 95% CI [0.97, 2.90]. Importantly, the interaction between rumination and depressive symptoms was significant, *B* = −0.93, *SE* = 0.35, Wald = 6.84, *p* = 0.009, OR = 0.40, 95% CI [0.20, 0.79]. Simple-slope analyses indicated that rumination was positively associated with current self-harm ideation at lower depressive symptom severity (−1 *SD*; OR = 4.23, 95% CI [1.48, 12.07], *p* = 0.007), whereas the association was not statistically significant at higher depressive symptom severity (+1 *SD*; OR = 0.66, 95% CI [0.34, 1.32], *p* = 0.24). The interaction pattern is presented in Figure 1.

As a supplementary analysis, a second binary logistic regression model was conducted to examine whether depressive symptoms also moderated the association between reflective thinking and current self-harm ideation. The overall model was statistically significant, χ^2^(3) = 30.39, *p* < 0.001, accounting for 16.9% of the variance in current self-harm ideation based on Cox and Snell *R*^2^ and 24.1% based on Nagelkerke *R*^2^. The Hosmer–Lemeshow goodness-of-fit test was non-significant, χ^2^(8) = 6.80, *p* = 0.558, indicating that the model demonstrated an adequate fit to the data. The interaction between reflective thinking and depressive symptoms was not significant, *B* = −0.11, *SE* = 0.22, Wald = 0.24, *p* = 0.626, OR = 0.90, 95% CI [0.59, 1.38], indicating no evidence that depressive symptoms moderated the association between reflective thinking and current self-harm ideation.

To examine whether the primary moderation finding was sensitive to dichotomization of self-harm ideation, an ordinal logistic regression was fitted using the original five-category outcome. The rumination × depressive symptom severity interaction remained negative and statistically significant (*B* = −0.830, *SE* = 0.334, OR = 0.44, 95% CI [0.23, 0.84], *p* = 0.013). A likelihood-ratio comparison with a multinomial logistic regression model did not detect a difference in fit, χ^2^(9) = 8.32, *p* = 0.503. The ordinal analysis yielded an interaction in the same direction as the primary binary model. However, the two highest response categories contained few participants, and the model-comparison result does not establish that the proportional-odds assumption holds.

A simulation-based sensitivity analysis indicated that, under the specified assumptions, the sample had approximately 80% power to detect a negative rumination × depressive symptom severity interaction coefficient of around −0.85 to −0.86 (corresponding OR ≈ 0.42–0.43). Estimated power was 79.7% at an assumed coefficient of −0.85 and 80.8% at −0.86, based on 10,000 simulations per effect size. The sample had lower sensitivity to smaller negative interactions. These estimates describe the detectable interaction magnitude under the simulation assumptions and do not constitute an a priori demonstration of sample-size adequacy.

## 4. Discussion

The present study examined emotional and cognitive correlates of current self-harm ideation among patients with a current or past major depressive episode. Guided by the theoretical frameworks of the emotional cascade model and response styles theory, the study aimed to clarify how depressive symptoms and repetitive cognitive processes jointly relate to self-harm ideation risk. Three key findings emerged from the analyses. First, participants reporting self-harm ideation exhibited more severe depressive symptoms and higher levels of rumination compared with those without such ideation. Second, rumination but not reflective thinking was found to be significantly associated with current self-harm ideation, whereas no significant association was detected for reflective thinking. Third, depressive symptom severity moderated the association between rumination and self-harm ideation, such that rumination was more strongly associated with self-harm ideation at lower levels of depressive symptoms.

### 4.1. Rumination and Current Self-Harm Ideation

The finding that rumination was associated with greater likelihood of self-harm ideation is consistent with theoretical and empirical research linking repetitive negative thinking to self-harm and suicide-related outcomes (Chapman et al., 2006; Lo & Cheng, 2024; Nicolai et al., 2016). According to response styles theory, rumination involves a repetitive and passive focus on the causes and consequences of one’s distress, which may maintain negative affect and interfere with effective problem solving. In this context, greater rumination may be associated with sustained emotional distress and maladaptive responses to distress, including self-harm ideation. However, given the cross-sectional nature of the present study, the direction of this association cannot be determined.

Empirical research provides further context for this association. A meta-analytic review found that greater rumination was associated with higher levels of suicidality, including suicidal ideation and suicide attempts (Rogers & Joiner, 2017). Prospective ecological momentary assessment research has also examined rumination as a potential proximal process linking stressful experiences with suicidal ideation. For example, Wallace et al. (2025) reported that rumination statistically mediated the association between daily negative life events and suicidal intensity among recently hospitalized adults. Importantly, these studies examined suicidal ideation and other suicide-related outcomes rather than the specific self-harm ideation outcome assessed in the present study. They therefore provide relevant contextual evidence for the observed association but do not establish that the same processes necessarily underlie self-harm ideation.

Within the framework of the emotional cascade model, rumination plays a central role in escalating cycles of emotional distress. The model proposes that rumination amplifies emotional responses, leading to increasingly intense negative affect that may become difficult to tolerate. In such circumstances, self-harm may emerge as a behavioral strategy to interrupt or escape this escalating cascade of rumination and emotional distress (Selby et al., 2013). Empirical studies applying this model have demonstrated that rumination is associated with both non-suicidal self-injury and heightened emotional reactivity, supporting the idea that repetitive negative thinking contributes to the escalation of distress preceding self-harm behaviors (Arbuthnott et al., 2015; Selby et al., 2009). However, the ECM’s account of self-harm primarily concerns the enactment of self-harm behavior and its proposed emotion-regulation function, whereas the present study assessed only current self-harm ideation. The observed association between rumination and self-harm ideation is therefore consistent with the broader theoretical relevance of rumination but does not establish that participants experienced an emotional cascade or engaged in self-harm behavior to regulate emotion.

### 4.2. Distinguishing Rumination from Reflective Thinking

In contrast to rumination, reflective thinking was not significantly associated with current self-harm ideation in the present study. This finding aligns with theoretical distinctions within response styles theory that differentiate maladaptive rumination from other forms of self-focused cognition (Treynor et al., 2003; Nolen-Hoeksema et al., 2008).

Empirical findings in the literature have suggested that reflection may show nonsignificant associations with suicidal outcomes compared with maladaptive rumination. For example, recent research examining cognitive processes associated with suicidality reported that reflective thinking showed statistically nonsignificant associations with suicidal ideation relative to rumination-related constructs (Horwitz et al., 2019). Taken together, these findings are consistent with the conceptual distinction between rumination and reflection as different forms of self-focused cognition. Although suicidal ideation and other suicide-related outcomes examined in prior research are not identical to self-harm ideation outcome assessed in the present study, that literature provides relevant context for considering their distinct associations. In the present sample, rumination was associated with self-harm ideation, whereas reflection was not significantly associated with it. The nonsignificant finding for reflection indicates insufficient evidence of an association in this sample and does not establish that reflection is adaptive or protective.

### 4.3. The Moderating Role of Depressive Symptom Severity

A central finding of the present study was that depressive symptom severity moderated the association between rumination and current self-harm ideation. Specifically, rumination was positively associated with self-harm ideation at lower depressive symptom severity, whereas its association was weaker and not statistically significant at higher depressive symptom severity.

One possible interpretation is that individual differences in rumination may be particularly relevant to self-harm ideation when depressive symptom severity is relatively low. According to response styles theory, ruminative responses may prolong negative mood and interfere with effective problem solving (Nolen-Hoeksema, 1991). The emotional cascade model (ECM) further proposes that rumination and negative affect can amplify one another (Selby et al., 2008). These theoretical processes offer a possible context for understanding why rumination was associated with self-harm ideation among individuals reporting less severe depressive symptoms. However, the ECM primarily addresses processes associated with self-harm behavior, and the present study did not directly assess emotional cascades or their temporal development.

At higher levels of depressive symptom severity, the association between rumination and self-harm ideation was weaker and not statistically significant. One possibility is that more pervasive distress may accompany self-harm ideation across a wider range of rumination levels, making differences in rumination less closely associated with whether ideation is reported. Taken together, the findings suggest that depressive symptom severity is an important context for understanding the association between rumination and current self-harm ideation: the strength of this association may not be uniform across individuals with a current or past major depressive episode. Future longitudinal research could examine the emotional processes associated with this variation and how these relationships unfold over time.

### 4.4. Implications for Clinical Practice

The present findings have several potential clinical implications. First, the results highlight rumination as a cognitive process that may contribute to current self-harm ideation among individuals with depression. Interventions that specifically target repetitive negative thinking, such as rumination-focused cognitive behavioral therapy or metacognitive approaches, may therefore hold promise for reducing self-harm ideation (Watkins & Roberts, 2020).

Second, the findings suggest that the association between rumination and current self-harm ideation varies according to depressive symptom severity. High levels of rumination may represent an important cognitive vulnerability factor (Nolen-Hoeksema, 1991). Rumination may partly reflect severity of depressive cognition rather than an entirely independent cognitive risk process. Identifying individuals who exhibit elevated rumination may allow clinicians to intervene earlier and potentially prevent further escalation into self-harm ideation.

### 4.5. Limitations and Strengths

Several limitations should be considered when interpreting the present findings. First, the cross-sectional design precludes any conclusions regarding the directionality or causality of the relationships among rumination, depressive symptoms, and self-harm ideation. Although the observed associations are consistent with theoretical perspectives discussed above, longitudinal research is needed to examine the temporal ordering of these variables and whether rumination is associated with subsequent changes in self-harm ideation.

Second, several statistical limitations warrant considerations. Dichotomizing the original five-category self-harm ideation item reduced the information available about variation among participants reporting ideation and may have affected statistical precision and effect estimates. Although an ordinal sensitivity analysis yielded an interaction in the same direction as the primary binary analysis, the two highest response categories contained few participants, limiting confidence in patterns across individual response categories. In addition, only 48 participants reported current self-harm ideation. A simulation-based sensitivity analysis indicated that, under the specified assumptions, the study had limited power to detect smaller interaction effects. Accordingly, the magnitude and precision of the observed moderation effect should be interpreted cautiously, and replication in larger clinical samples is warranted.

Third, the single self-harm ideation item did not distinguish thoughts involving suicidal intent from thoughts of non-suicidal self-injury. The focal constructs were assessed concurrently using self-report measures, which may introduce shared method variance. These limitations should be considered when interpreting the observed interaction.

Fourth, the sample included substantially more female than male participants. The small number of male participants limits the generalizability of the findings across genders and does not permit a reliable examination of gender differences in the observed associations, including the moderation effect. Replication in more gender-balanced samples is required.

Finally, the sample was recruited from a specific outpatient clinic setting, which may limit the generalizability of the findings to other clinical and community populations. Relevant clinical factors, including treatment and medication status, were not accounted for and may have influenced the observed associations. Future studies should assess these factors and consider their inclusion in analyses where theoretically and clinically appropriate. Replication in more diverse samples is therefore warranted.

Despite these limitations, the present study has several notable strengths. First, the use of a clinically diagnosed sample of individuals enhances the clinical relevance of the findings and extends research on rumination and self-harm outcomes to individuals receiving outpatient care for depression. Second, the study draws on the Response Styles Theory and the emotional cascade model to provide a theoretical context for examining the association between rumination and self-harm ideation, while recognizing that the emotional and behavioral processes proposed by these models were not directly tested. Third, by examining rumination and reflective thinking separately, the study offers a more differentiated account of their associations with current self-harm ideation. Finally, the examination of depressive symptom severity as a moderating factor suggests that the strength of the association between rumination and self-harm ideation may vary across levels of depressive symptom severity, providing a basis for further investigation in longitudinal clinical research.

## 5. Conclusions

In summary, the present study found that rumination was associated with current self-harm ideation among psychiatric outpatients with a current or past major depressive episode, whereas the association with reflective thinking was not statistically significant. Depressive symptom severity moderated the association between rumination and self-harm ideation, with a stronger positive association observed at lower levels of depressive symptoms. These findings are consistent with theoretical perspectives emphasizing the relevance of repetitive negative thinking in emotional distress and suggest that the strength of the association between rumination and self-harm ideation may vary according to depressive symptom severity. Longitudinal research is needed to determine whether changes in rumination are associated with subsequent changes in self-harm ideation.

## Figures and Tables

**Table 1:** Comparison of Demographic and Clinical Characteristics by Current Self-harm Ideation Status.

Variable	Overall (*N* = 164)		Self-Harm Ideation (*n* = 48)		No Ideation (*n* = 116)		*t*/χ^2^(df)	Effect Size	*p*
	*M*/*n* (%)	*SD*	*M*/*n* (%)	*SD*	*M*/*n* (%)	*SD*			
Gender							χ^2^(1) = 0.14	φ = 0.03	0.714
**Female**	136 (82.9)		39 (81.3)		97 (83.6)				
**Male**	28 (17.1)		9 (18.8)		19 (16.4)				
Age (years)	45.17	10.48	43.79	9.99	45.74	10.67	*t*(162) = 1.09	*d* = 0.19	0.280
HADS-D category							χ^2^(2) = 26.33	Cramér’s *V* = 0.40	<0.001
**Normal**	58 (35.4)		5 (10.4)		53 (45.7)				
**Borderline Abnormal**	43 (26.2)		11 (22.9)		32 (27.6)				
**Abnormal**	63 (38.4)		32 (66.7)		31 (26.7)				
HADS-D	9.15	4.36	11.94	3.54	8.00	4.15	*t*(162) = 5.76	*d* = 0.99	<0.001
DASS Total	51.27	27.71	72.96	22.88	42.14	24.34	*t*(160) = 7.49	*d* = 1.29	<0.001
**DASS Depression**	16.84	10.87	26.04	9.46	12.96	8.95	*t*(160) = 8.35	*d* = 1.44	<0.001
**DASS Anxiety**	15.01	9.28	20.67	7.92	12.63	8.80	*t*(160) = 5.46	*d* = 0.94	<0.001
**DASS Stress**	19.42	9.86	26.25	8.02	16.54	9.15	*t*(160) = 6.39	*d* = 1.10	<0.001
PSWQ Total	54.70	12.51	59.13	9.35	52.87	13.21	*t*(119.79) = 3.40	*d* = 0.51	<0.001
**RRQ Rumination**	42.54	7.93	45.73	5.45	41.22	8.42	*t*(132.89) = 4.07	*d* = 0.59	<0.001
**RRQ Reflection**	37.43	7.21	37.15	7.30	37.55	7.20	*t*(162) = 0.33	*d* = 0.06	0.744

Note. Continuous variables are presented as means (*M*) and standard deviations (*SD*). Categorical variables are presented as frequencies (n) and percentages (%). The *t*/χ^2^(df) column reports independent-samples t tests or Pearson chi-square tests, with degrees of freedom in parentheses. Effect sizes are reported as Cohen’s d, φ, or Cramér’s V, as appropriate. Valid sample sizes varied because of missing data. DASS = Depression Anxiety Stress Scales; HADS-D = Hospital Anxiety and Depression Scale–Depression subscale; PSWQ = Penn State Worry Questionnaire; RRQ = Rumination–Reflection Questionnaire.

**Table 2:** Spearman’s rho Correlations between Current Self-harm Ideation, Rumination, and Reflective Thinking.

Variable	1	2	3
1. Self-harm Ideation	—		
2. Rumination	0.268 ***	—	
3. Reflective thinking	− 0.041	0.246 ***	—

Note. *N* = 164. Self-harm was coded 0 = no, 1 = yes. Spearman’s rho coefficients are reported. *** *p* < 0.001.

**Table 3:** Logistic Regression Models Predicting Current Self-harm Ideation.

Predictor	*B*	*SE*	Wald	OR	95% CI	*p*
**Model 1** **. Rumination model**						
Rumination (z)	0.517	0.280	3.400	1.68	0.97–2.90	0.065
HADS-D (z)	1.360	0.316	18.513	3.90	2.10–7.24	<0.001
Rumination (z) × HADS-D (z)	−0.926	0.354	6.842	0.40	0.20–0.79	0.009
**Model 2. Reflection model**						
Reflective thinking (z)	−0.035	0.203	0.030	0.97	0.65–1.44	0.862
HADS-D (z)	1.074	0.224	22.991	2.93	1.89–4.54	<0.001
Reflection (z) × HADS-D (z)	−0.106	0.217	0.238	0.90	0.59–1.38	0.626

Note. Predictor variables were standardized prior to analysis. HADS-D = Hospital Anxiety and Depression Scale–Depression subscale; OR = adjusted odds ratio; CI = confidence interval.

## Data Availability

Data available from the corresponding author upon reasonable request due to privacy or ethical restrictions.

## Abbreviations

The following abbreviations are used in this manuscript:

DASS-21	21-item Depression Anxiety Stress Scales
DSM-5	Diagnostic and Statistical Manual of Mental Disorders, Fifth Edition
EAM	Experiential Avoidance Model
ECM	Emotional Cascade Model
HADS	Hospital Anxiety and Depression Scale
HADS-D	Hospital Anxiety and Depression Scale–Depression Subscale
MDD	Major Depressive Disorder
MDE	Major Depressive Episode
PSWQ	Penn State Worry Questionnaire
RRQ	Rumination–Reflection Questionnaire
SCID	Structured Clinical Interview for DSM Disorders
